# Kuhlmann vascularized bone grafting for treatment of Kienböck's disease: a case report

**DOI:** 10.11604/pamj.2016.24.37.8524

**Published:** 2016-05-10

**Authors:** Mohamed Ali Sbai, Hichem Msek, Sofien Benzarti, Monia Boussen, Riadh Maalla

**Affiliations:** 1 Orthopedics and Trauma Department, Maamouri Hospital, Nabeul, Tunisia; 2 Emergency Department, Mongi Slim hospital, La Marsa, Tunisia; 3 Plastic Surgery Department, Rabta Hospital, Tunis, Tunisia

**Keywords:** Kienböck disease, lunate, avascular necrosis, vascularized bone graft

## Abstract

Treatment of Kienböck's disease has historically been determined by staging, ulnar variance, and presence or absence of arthritic changes. With the advent of newer techniques of vascularized bone grafting, the status of the cartilage shell of the lunate has become another factor that can influence the procedure performed. The purpose of this article is to describe the technique of Kuhlmann vascularized bone graft for Kienböck's disease. In addition, the indications, contraindications, and outcomes are described.

## Introduction

Described in 1910 by Robert Kienböck, Kienböck's disease is an unusual problem that causes osteonecrosis and collapse of the lunate bone, which leads to chronic dysfunction and pain. It is usually seen in adults between 20 and 40 years of age and is typically unilateral [1, 2]. Despite its long lasting recognition, the etiology and natural history of this disease remain unknown at present and multifactorial pathogenesis which is influenced by mechanical, anatomic, vascular, traumatic, and systemic factors has been proposed. Kienböck's disease displays a progressive nature and destructs the wrist joint, so prompt diagnosis and appropriate treatment are obligatory [3, 4]. There is many therapeutic choices in Kienböck's disease, varied and often controversial and indications are guided by the stage of the disease. The objective of this work is to show the interest of the Kuhlmann vascularized bone graft in support of Kienböck's disease [2, 3].

## Patient and observation

**Figure 1:**
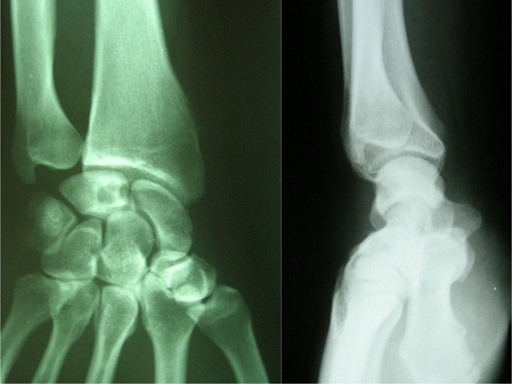
Preoperative radiogram of a 40-year-old male with Lichtman type II Kienböck's disease

**Figure 2:**
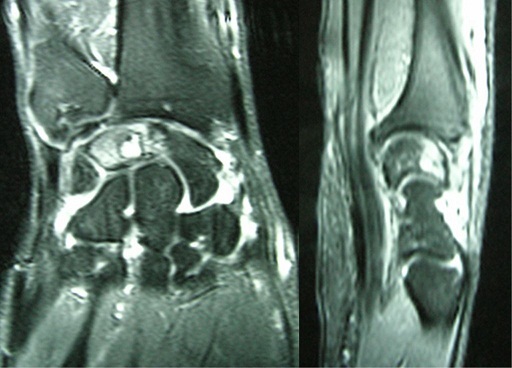
MRI, T2-weighted image and MRI arthrogram

**Figure 3:**
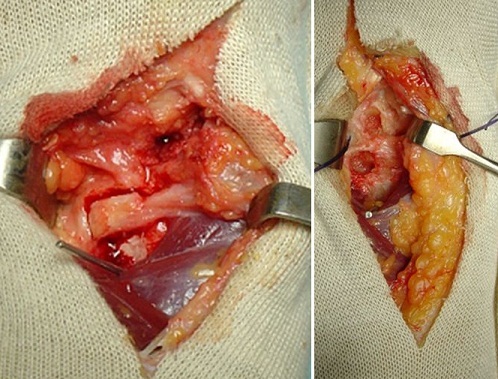
Per operative volar radial vascular bone grafting procedure

**Figure 4:**
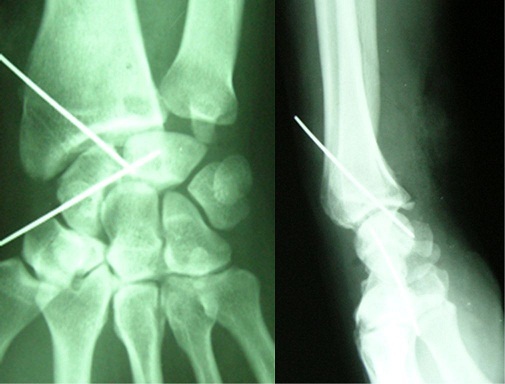
Postoperative radiogram: the lunate stabilized by two pins

**Figure 5:**
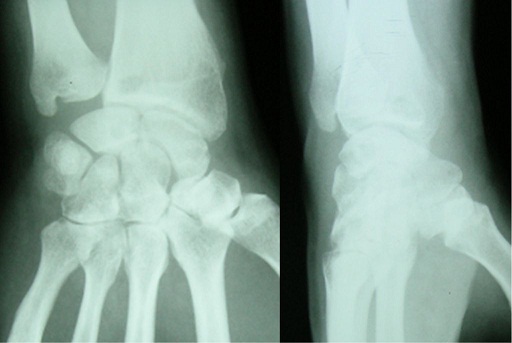
Radiographs showed consolidation after three months

We present the case of a 40-years-old patient, a rightly construction worker who consults for back pain in the right wrist, occurring in manual efforts and partnering intermittently at discrete effusions to spontaneous resolution. Clinical examination revealed pain on palpation of the dorsal skin projection of the lunate (depression in the base of the third metacarpal) without repercussion on the amplitude of the wrist movements. The radiographs and MRI for an II staged Kienböck Lichtman disease (Figure 1, Figure 2). Furthermore, the ulnar variance (VU = distance between the distal articular surface of the radius and ulna) is zero. Thus, it was decided to opt for this patient an excision of the necrotic part of the lunate by filling with vascularized bone graft taken at the distal radius: Based on the work of Kuhlman, the graft is taken from the anterior surface of the radius, vascularized by the anterior carpal artery, whose constant artery pedicle is long enough to reach the scaphoid as well as lunate. The surgical approach used is a conventional route of Henry with an internal hook for enlarging the incision opening the carpal tunnel. Before withdrawing the graft, the lunate is prepared to determine the size of the defect (Figure 3), the graft is then opened up to the front side of the radius. Bone graft is then placed so as to fill the defect on the lunate and stabilized by two pins (scapholunate and radio-lunar), (Figure 4). The closure is done on a Redon drain. A front brace leaving free the elbow and 40^°^ with the wrist extension is positioned to consolidation. This comfortable functional position for the patient is permitted by the length of the pedicle. The pin was kept up consolidation, it was found on the 45th postoperative day. The consolidation with integration of the graft was seen on radiographs performed on the 45^th^ postoperative day. The mobility of the wrist flexion/extension, pronation/ supination after rehabilitation is comparable to the controlateral side at the 90th postoperative day. The net decrease in pain and progressive authorized the resumption of work but with caution to avoid tools with vibration (Figure 5).

## Discussion

The treatment of this disease is a challenging problem. The goals of treatment include maintaining normal wrist kinematics, conservation of wrist function, and, when and if possible, revascularization of the necrotic bone. Several methods have been suggested in the literature for treatment of Kienböck's disease, but, based on previous data, no active treatment is superior in the treatment of this disease and there are neither homogeneous studies nor large series that have described which treatment is best. Treatment modalities range from conservative methods such as immobilization to operative ones such as intercarpal fusions, excision, partial capitate shortening, implant arthroplasty, ulnar lengthening, radial shortening, and vascularized bone grafting [5]. When either of the conservative methods is unsuccessful or there is a more advanced stage, surgical procedure is recommended. In Lichtman stage II of disease, sclerosis of the lunate and/or early collapse of the lunate radial border may appear. In stage IIIA, there is more severe collapse. In stages II and IIIA, the remainder of the carpus is still uninvolved, so treatment involves surgeries for revascularization of the lunate either directly (with vascularized bone grafting) or indirectly (by unloading the lunate) [6]. Distal radial shortening is one of the most commonly used procedures and recent reports of long-term outcomes of radial shortening osteotomy for earlier stages have shown that this method is a reliable treatment option for relieving pain and improving function [7]. Since the most apparent predisposing factor of disease is the unsteady blood supply to the lunate, newer modalities such as vascularized bone grafts, which improve direct lunate revascularization, may arrest the progression of collapse. However, current data are not enough to conclude with certainty whether this type of surgeries shows more improvement over the traditional treatment choices [6]. Some studies represented that there is no substantial clinical difference between the radial shortening and vascularized bone graft in long-term outcome; but some suggested that decompression surgery along with revascularization may provide better results than either alone [8].

## Conclusion

The use of bone graft taken from the front face level of the radius and vascularized by the volar carpal artery is a good treatment of the disease stages II of Lichtman. It could be extended to stage IIIA and join a radius shortening osteotomy in case of short ulna. If the levy graft may seem a bit difficult the first few times, it's actually a simple technique which has given excellent results since our consolidation period is 45 days. The use of a single anterior approach allows at the same time operative general anesthesia and hospitalization. If this technique was first described for the treatment of conventional techniques failures, the quality of functional results and consolidation of speed did offer us as primitive treatment of stage II and IIIA Lichtman.
